# Short-Term Effects of Thoracic Spine Manipulation on the Biomechanical Organisation of Gait Initiation: A Randomized Pilot Study

**DOI:** 10.3389/fnhum.2017.00343

**Published:** 2017-06-30

**Authors:** Sébastien Ditcharles, Eric Yiou, Arnaud Delafontaine, Alain Hamaoui

**Affiliations:** ^1^CIAMS, Université Paris-Sud, Université Paris-SaclayOrsay, France; ^2^CIAMS, Université d’OrléansOrléans, France; ^3^Ecole Nationale de Kinésithérapie et Rééducation (ENKRE)Saint-Maurice, France; ^4^Laboratoire de Physiologie de la Posture et du Mouvement (PoM Lab), Université JF ChampollionAlbi, France; ^5^Laboratoire Activité Physique, Performance et Santé (MEPS), Université de Pau et des Pays de l’Adour (UPPA)Tarbes, France

**Keywords:** anticipatory postural adjustments, gait initiation, spinal manipulation HVLA, T9 vertebrae, range of motion, posturo-kinetic capacity

## Abstract

Speed performance during gait initiation is known to be dependent on the capacity of the central nervous system to generate efficient anticipatory postural adjustments (APA). According to the posturo-kinetic capacity (PKC) concept, any factor enhancing postural chain mobility and especially spine mobility, may facilitate the development of APA and thus speed performance. “Spinal Manipulative Therapy High-Velocity, Low-Amplitude” (SMT-HVLA) is a healing technique applied to the spine which is routinely used by healthcare practitioners to improve spine mobility. As such, it may have a positive effect on the PKC and therefore facilitate gait initiation. The present study aimed to investigate the short-term effect of thoracic SMT-HVLA on spine mobility, APA and speed performance during gait initiation. Healthy young adults (*n* = 22) performed a series of gait initiation trials on a force plate *before* (“pre-manipulation” condition) and *after* (“post-manipulation” condition) a sham manipulation or an HVLA manipulation applied to the ninth thoracic vertebrae (T9). Participants were randomly assigned to the sham (*n* = 11) or the HVLA group (*n* = 11).The spine range of motion (ROM) was assessed in each participant immediately after the sham or HVLA manipulations using inclinometers. The results showed that the maximal thoracic flexion increased in the HVLA group after the manipulation, which was not the case in the sham group. In the HVLA group, results further showed that each of the following gait initiation variables reached a significantly lower mean value in the post-manipulation condition as compared to the pre-manipulation condition: APA duration, peak of anticipatory backward center of pressure displacement, center of gravity velocity at foot-off, mechanical efficiency of APA, peak of center of gravity velocity and step length. In contrast, for the sham group, results showed that none of the gait initiation variables significantly differed between the pre- and post-manipulation conditions. It is concluded that HVLA manipulation applied to T9 has an immediate beneficial effect on spine mobility but a detrimental effect on APA development and speed performance during gait initiation. We suggest that a neural effect induced by SMT-HVLA, possibly mediated by a transient alteration in the early sensory-motor integration, might have masked the potential mechanical benefits associated with increased spine mobility.

## Introduction

The coordination between posture and movement is known to be a key factor in motor performance. Gait initiation, which is the transient phase between quiet standing posture and ongoing walking, is a classical model used in the literature to investigate this coordination (e.g., Mann et al., 1979; Brenière et al., 1987; Yiou et al., 2007; Honeine et al., 2016). It is composed of a postural phase preceding the swing foot-off, which corresponds to the “anticipatory postural adjustments (APA)”. The postural phase is followed by an execution phase ending when the peak of forward center of gravity (COG) velocity (or speed performance) is reached (Brenière et al., 1987; Lepers and Brenière, 1995). During these APA, the forward propulsive forces required to reach the peak COG velocity are generated by an anticipatory backward center of pressure (COP) shift. The larger this shift, the greater the speed performance (Brenière et al., 1987; Lepers and Brenière, 1995). The relationship between APA and speed performance during gait initiation illustrates the biomechanical concept of “Posturo-kinetic capacity (PKC)” (Bouisset and Zattara, 1987; Bouisset and Do, 2008), according to which the motor performance of any motor task (in terms of speed, force or precision) depends on the capacity of the central nervous system to generate appropriate APA. According to this concept, any factors that would impair (or conversely enhance) APA development may impair (or enhance) the motor performance. This PKC concept was substantiated by experimental studies which investigated the relationship between postural chain mobility, APA and motor performance during various motor tasks such as isometric ramp push (Le Bozec and Bouisset, 2004), pointing (Lino et al., 1992; Teyssèdre et al., 2000), and more recently, trunk flexion (Diakhaté et al., 2013) or sit-to-stand (Diakhaté et al., 2013; Alamini-Rodrigues and Hamaoui, 2016; Hamaoui and Alamini-Rodrigues, 2017a,b). In these studies, postural chain mobility was varied by changing the seat-thigh contact (Teyssèdre et al., 2000; Le Bozec and Bouisset, 2004; Diakhaté et al., 2013), by increasing the muscular tension along the torso (Hamaoui et al., 2004, 2011; Hamaoui and Le Bozec, 2014) or by restraining the spine mobility at different levels by means of splints (Alamini-Rodrigues and Hamaoui, 2016; Hamaoui and Alamini-Rodrigues, 2017a,b). These studies showed that the restriction of the postural chain mobility, and especially the spine mobility, has a negative influence on APA and motor performance. Conversely, according to the PKC concept, enhancing the postural chain mobility should have a positive influence on these parameters. Besides this purely mechanical influence, APA associated with stepping initiation are also known to be finely tuned to the continuous proprioceptive (Ruget et al., 2010) and cutaneous inflow (Do and Gilles, 1992; Ruget et al., 2008) arising from the postural body segments. Perturbations of this sensory inflow, e.g., by reducing the plantar support or by vibrating the ankle muscles, have been shown to alter APA and motor performance.

“Spinal Manipulative Therapy High-Velocity, Low-Amplitude” (SMT-HVLA) is a healing technique applied to the spine that has been used for centuries by healthcare practitioners including Osteopaths, Chiropractors and Physiotherapists to relieve symptomatic patients from acute and chronic low back/neck pain and/or to improve spine mobility (Wiese and Callender, 2005). As such, SMT-HVLA may have the potential to improve the PKC and thus motor performance. As stressed in the literature (e.g., the review of Pickar and Bolton, 2012), a number of sustained changes in the spinal biomechanics have been thought to occur as a result of SMT-HVLA. For example, the impulsive thrust delivered during the manipulation may alter the segmental biomechanics by releasing trapped meniscoïds, releasing adhesions, or by diminishing distortion in the intervertebral disc. In addition, recent studies reported relaxation of paraspinal muscles following SMT-HVLA as revealed with decreased electromyographic (EMG) activity (DeVocht et al., 2005; Lehman, 2012). Increased spine mobility might result from such changes in the spinal biomechanics and/or EMG activity. Interestingly, this technique is nowadays widely used by healthy athletes (runners, footballers, sprinters etc.) just before a competition in order to reach their “peak performance” (Leonardi, 1994). However, it must be noted that the effect of SMT-HVLA on the articular free play is still controversial (for review see Millan et al., 2012a), with mitigated results on sports performance (Miners, 2010). Shrier et al. (2006) compared jump height and running velocity with and without pre-event SMT-HVLA in elite healthy athletes. These authors found that there was no significant effect of SMT-HVLA on the countermovement jump height and sprint times. However, they also stressed that the direction and magnitude of the observed changes were consistent with a clinically relevant performance enhancement. A similar conclusion was stated by Humphries et al. (2013) with regard to the immediate effect of lower cervical spine manipulation on handgrip strength and free-throw accuracy of asymptomatic basketball players. These authors reported a slight increase in free-throw percentage, which according to them, deserved further investigation.

Besides the potential increase in spine mobility, movement kinematics may also be potentially influenced by neurophysiological changes induced by SMT-HVLA. For example, studies on the anesthetized cat have shown that spinal manipulation induced changes in the discharge of somatosensory afferents from the paraspinal region (Pickar, 2002; Pickar and Bolton, 2012; Reed et al., 2015), including those afferents innervating muscle spindles, Golgi Tendon Organs and high threshold mechanoreceptors. There are currently no unequivocal data regarding whether SMT-HVLA activates nociceptors. In humans, changes in the sensori-motor pathways following SMT-HVLA have been reported in the literature, but with sometimes contradictory results. For example, studies using the Hoffman reflex (H-reflex) technique indicated that spinal manipulation induced a decreased motoneuronal excitability in asymptomatic subjects (Murphy et al., 1995; Dishman and Burke, 2003) and in low back pain patients (Suter et al., 2005), while Niazi et al. (2015) indicated, on the contrary, an increased excitability. Data collection and data analysis methodology of the H-reflex have been evoked by these latter authors to explain this discrepancy with the literature. At the cortical level, it seems that there exists a consensus concerning the alteration of the sensorimotor processing and sensorimotor integration following spinal manipulation, as evidenced with the somatosensory-evoked potential technique (e.g., Haavik-Taylor and Murphy, 2007; Taylor and Murphy, 2008; Haavik Taylor and Murphy, 2010; see “Discussion” Section on this aspect).

As stressed in the literature (e.g., Pickar and Bolton, 2012), the extent to which these mechanical and neurophysiological responses to spinal manipulation reflect beneficial outcomes (e.g., pain relief or enhanced spine mobility) remains unclear. However, each of these responses has the potential to induce changes in the coordination between posture and movement, which strongly relies on both sensory inputs from the postural limbs and postural joint mobility as stressed above. The present study, therefore, aimed to investigate the short-term effect of SMT-HVLA on spine mobility, APA and speed performance during gait initiation in young healthy adults. We first hypothesized that a SMT-HVLA applied to the ninth thoracic vertebra (T9) will increase the spine range of motion and facilitate APA development in the gait initiation paradigm, which is known to involve spine mobility (e.g., Ceccato et al., 2009). Second, we also assumed that the various short-term neurological effects of this manipulation may either improve or reduce the PKC and task performance.

## Materials and Methods

### Subjects

**Figure 1:**
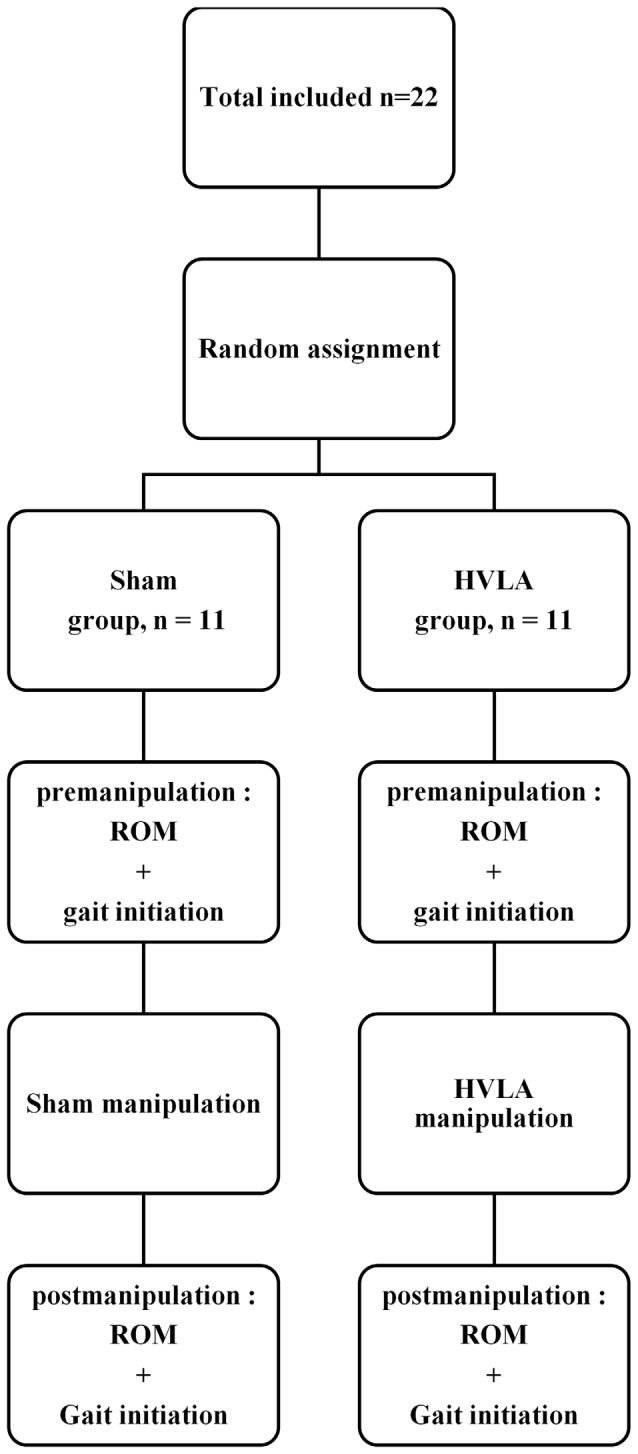
Study flow diagram.

The study was a randomized investigation that included 22 right-handed young healthy adults. The non-probability convenience method was used, i.e., participants were randomly assigned to one of the two following groups using the envelope method (Figure 1): 11 participants (six female, five male; 28 ± 4 years [mean ± SD]; 64 ± 8 kg; 169 ± 8 cm) were assigned to the HVLA group and eleven participants (five female, six male; 29 ± 4 years; 63 ± 8 kg; 170 ± 8 cm) to the sham group. Participants were blinded to their group allocation. They had no known contraindications to spinal manipulation such as recent history of trauma, known metabolic disorders, inflammatory infectious arthropathies, or bone malignancies. None of them suffered from back pain during the experiment or have suffered in the past months. In addition, participants were all naïve about SMT-HVLA manipulation. They all gave written consent after having been informed of the nature and purpose of the experiment which was approved by local ethics committees from the CIAMS Research Unit, Equipe d’Accueil (EA) 4532. The study complied with the standards established by the Declaration of Helsinki. Our study was assigned the following trial registration number: 2017-002389-34.

### Experimental Task and Conditions

All experiments took place in the Biomechanics laboratory of the Paris Saclay University which is located within the Kremlin Bicêtre Hospital (Paris, France). Physical conditions (room temperature and time of the day) were common to all treatment groups (see below), and also constant before/after the manipulation.

Participants initially stood barefoot in a natural upright posture on a force plate embedded at the beginning of a 6 m walkway track. The feet were shoulder-width apart, with the arms alongside the trunk and the gaze directed forward to a small target at eye level and out of reach (2 cm diameter, 5 m distant). The locations of the heel and big toe of each foot in the initial posture were marked with sections of adhesive tape placed on the force plate and were used as a visual reference on which participants positioned themselves under the supervision of the experimenters. From the initial posture, participants performed two series of ten gait initiation trials: one just before, and a second one immediately after a specific manipulation (pre- and post-manipulation conditions, respectively) depending on their group (HVLA or sham). All ten trials within each condition were averaged. In these two conditions, participants initiated gait at a spontaneous velocity and at their own initiative following an auditory signal delivered by the experimenter, and then continued walking straight until the end of the track. Participants initiated gait with their preferred leg in all trials. One blank trial was provided in the pre-manipulation condition (not recorded) to ensure that the instructions were well understood by the participant and that the material was operational. The rest time was approximately 10 s between trials. The Range of Motion (ROM) of the thoracic spine was assessed (see description below) for each participant in the HVLA and sham groups immediately before and after the HVLA or sham manipulations (see description below), respectively.

### HVLA and Sham Manipulations

The HVLA and sham manipulations complied with the 2016 Consensus on Interventions Reporting Criteria List for Spinal Manipulative Therapy (CIRCLE SMT; Groeneweg et al., 2017). Both manipulation procedures were performed by one of the authors of the present study, an experienced professional physiotherapist and osteopath practitioner with 10 years of clinical experience in his own practice. The practitioner is also a teacher at the Ecole Nationale de Kinésithérapie et Rééducation (France). He has received extensive training in the study protocols and was certified for both thoracic lift manipulation and sham procedure by simulating multiple study visit scenarios overseen by research team members.

SMT-HVLA was applied to the ninth thoracic vertebra (T9) since this vertebra is described as the “walking vertebra”, a concept arising from the classical article of Wernham (1985). This concept is based on the fact that the T9 vertebra is the inflexion point of the curvature change of thoracic cyphosis in lumbar lordosis. This vertebra ensures the junction between the thoracic and lumbar segments, mainly in their counter-rotation movement, especially during walking. In this plane, the center of rotation between the thoracic and the pelvic belts is presumably positioned between L3 and T7 (Konz et al., 2006).

In the HVLA group, the spinous process of the ninth thoracic vertebrae (T9) of the participant was identified by the practitioner and was marked with a pen. The participant stood upright with the hands positioned on the transverse processes of the selected vertebra with palms facing the back. The practitioner stood behind him/her with the front foot positioned between the participant’s feet. The practitioner circled the participant’s trunk by passing his arms under his/her armpits, and his chest was in contact with the palms of the participant’s hands. From this posture, he applied a single manual rapid horizontal pressure to the T9 vertebrae, followed by a single rapid vertical traction of the vertebral column. This technique corresponds to the standing thoracic “lift-off” technique. Before the manipulation, the practitioner systematically informed the participant that the sound of a cavitation was not a sign of success, and after the manipulation, that the manipulation was successful.

In the sham group, the experimental protocol was exactly the same as in the HVLA group with regard to the T9 marking, the initial/final postures, and the information given to the participant on the efficacy of the manipulation (positive verbal reinforcement). This guaranteed the blindness of participants with respect to their group allocation. Only the manipulation differed between the two groups. The manipulation used in the sham group corresponded to the “light touch methodology” validated by the North Texas Chronic Low Pain Trial (Licciardone et al., 2013). In this manipulation, the practitioner did not apply any compression or traction of the vertebral column but solely maintained the above-described posture with the participant for 10 s.

**Figure 2:**
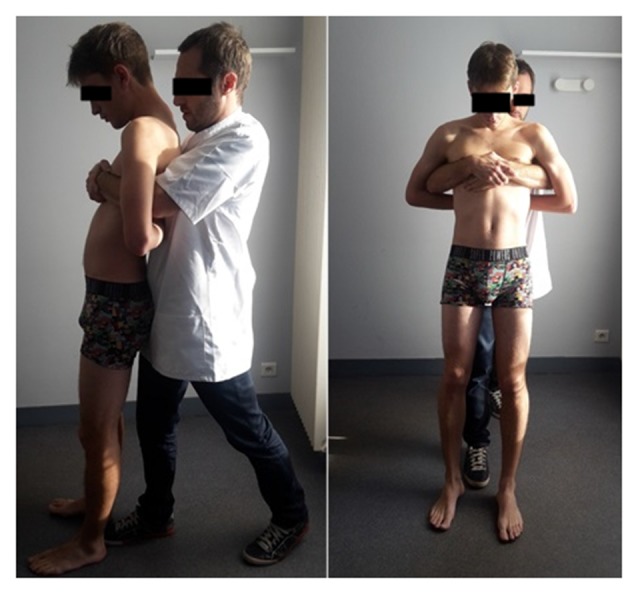
Profile (left) and front (right) views of the participant’s and the practitioner’s initial postures in the sham and HVLA manipulations. The written informed consent was obtained from the participants depicted in the images.

The HVLA and sham manipulations took place beside the force plate to ensure minimal time between the end of the manipulation procedure and the beginning of the first gait initiation trial of the post-manipulation condition. A brief overview of the practitioner’s and the participant’s postures adopted for the manipulations is provided in Figure 2.

### Evaluation of Spine ROM

**Figure 3:**
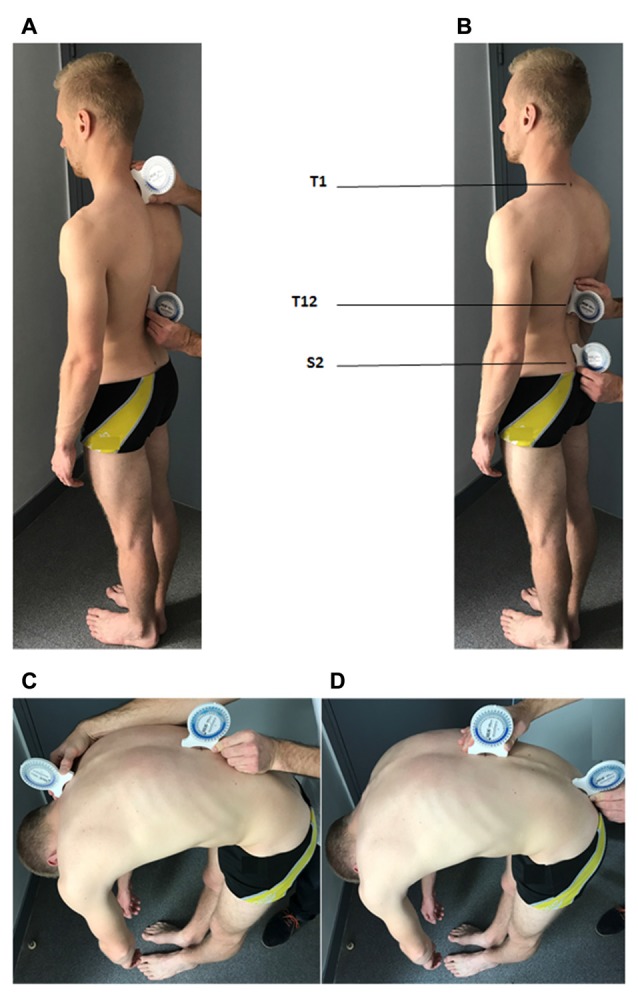
Positioning of the two inclinometers for the evaluation of the spine range of motion. **(A)** Inclinometers are positioned over the spinous process of the first and last thoracic vertebrae (T1 and T12, respectively) and are calibrated in this position for the evaluation of the maximal thoracic flexion **(C)**. **(B)** Inclinometers are positioned over T12 and the spinous process of the second sacral vertebrae (S2) and are calibrated in this position for the evaluation of the maximal lumbar flexion **(D)**. The written informed consent was obtained from the participants depicted in the images.

Spine ROM was evaluated before the series of gait initiation trials in the pre- and post-manipulation conditions (Figure 1). Two inclinometers (Bubble^®^ Inclinometer, Fabrication Enterprises, White Plains, NY, USA) were used to evaluate spine ROM. The reliability and accuracy of inclinometers in measuring lumbar lordosis and cervical spine flexion and extension ROM have been assessed in previous studies (Lewis and Valentine, 2010; Garmabi et al., 2012). The measurement of the spine ROM was conducted according to the standard protocol set out in the American Medical Association guide to the evaluation of permanent impairment (Doege and Houston, 1993; Cocchiarela and Andersson, 2001). The spinous process of the first and last thoracic vertebrae (T1 and T12) and the second sacral vertebrae (S2) of the participant were identified by the experimenter and marked with a pen while the participant stood upright. The inclinometers were then placed on these marks two by two (T1 and T12 or T12 and S2) and were calibrated to zero in this position. The participant was then instructed to perform maximum trunk flexion and extension with legs stretched. Each movement was repeated two times with the inclinometer positioned at T1/T12 then at T12/S2 (Figure 3). The mean ROM value obtained in these two trials was computed. For each movement direction, trunk inclination was computed as the difference between the values provided by the two inclinometers (thoracic flexion/extension: T1/T12; lumbar flexion/extension: T12/S2). The thoraco-lumbar flexion and extension were calculated from the sum of the thoracic and lumbar values in flexion and extension, respectively.

### Materials

External forces and moments applied to the participants were recorded from a force plate (600 × 1200 mm, AMTI, Watertown, MA, USA). Before analysis, the force-plate signals were filtered using a low-pass Butterworth filter with a 10 Hz cutoff frequency (Caderby et al., 2017). Biomechanical data were sampled at 500 Hz and stored on a hard disk for off-line analysis. Data acquisition and stimulus display were controlled by a custom-made program written in Matlab^TM^ (R2009b, The MathWorks Inc., Natick, MA, USA). Only the postural dynamics along the anteroposterior axis were considered in the present study as we were mainly interested in the speed performance of gait initiation. Instantaneous COG acceleration was obtained with the ratio [ground reaction forces/subject’s mass] following Newton’s second law (ΣF = mγ, where ΣF, the sum of external forces applied to the whole body; m, body mass; γ, COG acceleration). The COG velocity was obtained through simple integration of the COG acceleration trace. The instantaneous COP displacement (xP) was computed using the formula:

$$\text{xP}=\frac{-\text{My}+\text{Fx}\times d\text{z}}{\text{Fz}}$$

where My, Fx, Fz are the moment around the mediolateral axis, the anteroposterior and vertical ground reaction forces, respectively; *d*z is the distance between the surface of the force plate and its origin, located at the center of the force plate.

**Figure 4:**
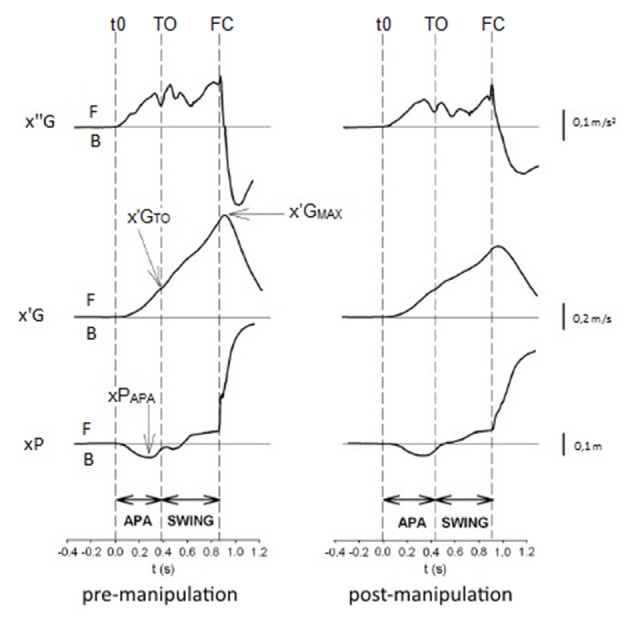
Biomechanical profiles of gait initiation in the pre- and post-manipulation conditions in one representative participant of the HVLA group. x″G, x′G, xP: anteroposterior center of gravity (COG) acceleration, COG velocity, and center of pressure (COP) displacement, respectively. t0, TOT, foot-contact (FC): onset rise of x″G trace, swing toe-off (TO) and swing FC. Anticipatory postural adjustments (APA), SWING: APA and swing phase. x′G_MAX_, x′G_TOT_, xP_APA_: peak of COG velocity, COG velocity at TO, peak of anticipatory backward COP displacement. F: forward displacement, velocity or acceleration, B: backward displacement, velocity or acceleration.

Swing toe-off (TO) and foot-contact (FC) instants were detected with force plate data (Caderby et al., 2013) and with foot switches (Force Sensing Resistor, 1 cm^2^ surface, Biometrics, France) affixed under the heel and big toe of the swing foot. The “biomechanical traces” (see Figure 4) will refer to the COP displacement and COG velocity traces obtained from the force plate recordings.

### Gait Initiation Variables

Data acquisition was triggered 200 ms prior to the auditory signal, which allowed *post hoc* calculation of the COP position in the initial posture. The anteroposterior component of the COP initial position was computed as the mean COP value obtained during these 200 ms. APA duration corresponded to the time between the onset rise of the COP trace to the time of swing TO (e.g., Yiou et al., 2011; Delafontaine et al., 2015). The APA onset was detected when the COP trace deviated 2.5 standard deviations from its baseline value (e.g., Caderby et al., 2017). APA amplitude was estimated with the forward COG velocity at the TO time and with the maximal backward COP displacement during APA (Figure 4). Swing phase duration corresponded to the time between swing TO and swing FC. Step motor performance (speed performance) was quantified with the maximal COG velocity. Step length corresponded to distance between the peak backward COP position obtained during the APA and the COP position at the time of the rear TO (Yiou et al., 2016). Finally, the mechanical efficiency of the APA was quantified with the ratio [COG velocity at TO/APA duration] (Yiou et al., 2011). It is assumed that the greater this ratio, the greater the mechanical efficiency.

The experimenter who analyzed the data and performed the ROM measures (pre and post treatment) was different from the practitioner and was blinded to the treatment group so as to ensure absence of expectation bias and optimize the reliability of the test procedure.

### Statistics

Mean values and standard deviations of ROM and gait initiation variables were computed in each condition for all subjects. The normality of data was checked using the Kolmogorov-Smirnov test and the homogeneity of variances was checked using the Bartlett test. A 2 × 2 mixed-model analysis of variance (ANOVA) was used, with GROUP (HVLA vs. sham) as the between-subject factor and CONDITION (pre-manipulation vs. post-manipulation) as the within-subject factor. For each ANOVA, the hypothesis of interest was the 2-way-interaction (GROUP × CONDITION). Significant outcomes were followed up with the Tukey *post hoc* test. In addition, the participants’ anthropometrical characteristics were compared between groups using independent Student’s *t*-tests for continuous data, and chi-square tests of independence were used for categorical data to evaluate the adequacy of the randomization. The level of statistical significance was set at alpha = 0.05. Data analysis was performed using Statistica 12, statsoft^®^.

## Results

### Anthropometrical Characteristics of Participants

Participants were randomly assigned to the sham or HVLA groups. Their anthropometrical characteristics are reported in Table 1. Statistical analysis showed that the two groups were homogenous in terms of mean age, gender, height and weight.

**Table 1:** Anthropometrical characteristics of participants.

	HVLA group (*n* = 11)	Sham group (*n* = 11)	*P* Value
Age (years)	28 ± 4	29 ± 4	0.633^†^ NS
Gender	Females 6	Females 5	0.670^‡^ NS
	Males 5	Males 6	
Height (cm)	169 ± 8	170 ± 8	0.913^†^ NS
Weight (kg)	64 ± 8	63 ± 8	0.815^†^ NS

*Values given are means ± 1 standard deviation, except for gender; ^†^Independent samples t test; ^‡^Chi-square test. NS: non-significant difference*.

### Comparison of Spine ROM between Groups and Conditions

The results showed that there was no significant main effect of GROUP, CONDITION or GROUP × CONDITION interaction on any of the spine ROM values, except on the thoracic flexion. For this variable, there was a significant main effect of GROUP (*F*_(1,21)_ = 4.53, *p* < 0.05), CONDITION (*F*_(1,21)_ = 15.73, *p* < 0.01) and GROUP × CONDITION interaction (*F*_(1,21)_ = 14.55, *p* < 0.01). For the HVLA group, the *post hoc* analysis further indicated that this variable was significantly larger in the post-manipulation condition (mean value: 24 ± 12°) than in the pre-manipulation condition (20 ± 12°) (*p* < 0.05). In contrast, for the sham group, it was not significantly different. Finally, it is noteworthy that there was no significant difference in any of the spine ROM values (including the thoracic flexion) between the HVLA and the sham group in the pre-manipulation condition. The spine mobility was therefore equivalent between the two groups before the manipulation.

### Description of Typical Biomechanical Traces Obtained during Gait Initiation

The time-course of the biomechanical traces obtained during gait initiation was globally similar in the pre- and the post-manipulation condition for both the HVLA and sham groups. As classically reported in the literature, the swing TO was systematically preceded by dynamic phenomena corresponding to APA (Figure 4). These APA included the backward COP displacement along with the forward COG acceleration. The COG velocity increased progressively until it reached a maximum value a few milliseconds after the time of swing FC.

### Comparison of Gait Initiation Variables between Groups and Conditions

The results showed that there was a significant main effect of GROUP on every gait initiation variables investigated in this study, i.e., APA duration (*F*_(1,21)_ = 6.25, *p* < 0.01), peak of anticipatory backward COP displacement (*F*_(1,21)_ = 19.07, *p* < 0.001), COG velocity at TO (*F*_(1,21)_ = 6.92, *p* < 0.01), mechanical efficiency of APA (*F*_(1,21)_ = 10.05, *p* < 0.01), peak COG velocity (*F*_(1,21)_ = 19.75, *p* < 0.001), step length (*F*_(1,21)_ = 11.81, *p* < 0.001) and swing phase duration (*F*_(1,21)_ = 5.87, *p* < 0.01). In addition, there was a significant main effect of CONDITION on each of the following variables: APA duration (*F*_(1,21)_ = 3.95, *p* < 0.05), peak of anticipatory backward COP displacement (*F*_(1,21)_ = 19.73, *p* < 0.001), COG velocity at TO (*F*_(1,21)_ = 12.40, *p* < 0.001), mechanical efficiency of APA (F_(1, 21)_ = 9.39, *p* < 0.01), peak COG velocity (*F*_(1,21)_ = 12.04, *p* < 0.001) step length (*F*_(1,21)_ = 22.22, *p* < 0.001) and swing phase duration (*F*_(1,21)_ = 2.39, *p* < 0.05). Finally, there was a significant GROUP X CONDITION interaction on each of the following variables: APA duration (*F*_(1,21)_ = 2.92, *p* < 0.05), peak of anticipatory backward COP displacement (*F*_(1,21)_ = 11.92, *p* < 0.01), COG velocity at TO (*F*_(1,21)_ = 4.22, *p* < 0.05), mechanical efficiency of APA (*F*_(1,21)_ = 8.51, *p* < 0.01), peak COG velocity (*F*_(1,21)_ *=* = 3.27, *p* < 0.05), step length (*F*_(1,21)_ = 9.66, *p* < 0.01) and swing phase duration (*F*_(1,21)_ = 6.29, *p* < 0.05).

**Figure 5:**
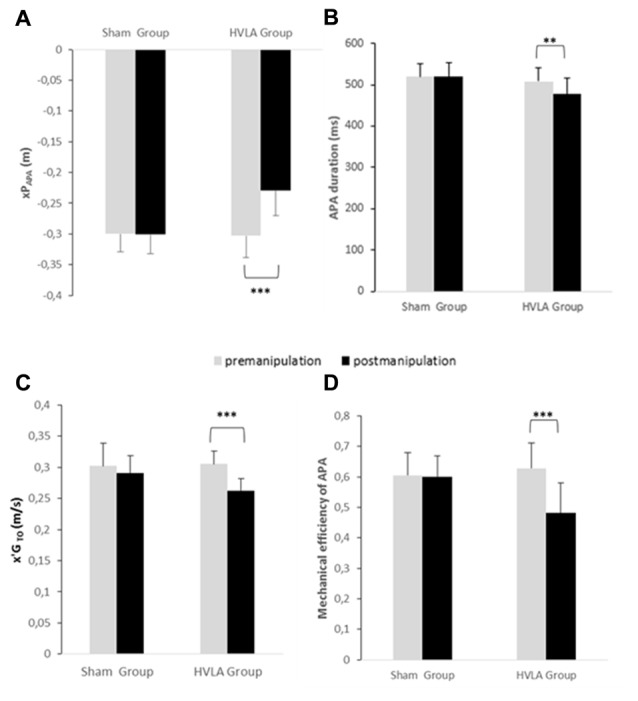
Comparison of APAs related variables between the pre- and post-manipulation conditions in the sham and HVLA groups. **(A)** xP_APA_: peak of anticipatory backward center of pressure displacement, **(B)** APA duration, **(C)** x′G_TOT_: center of gravity velocity at TO and **(D)** mechanical efficiency of APAs. **, *** Statistical difference with *p* < 0.01, *p* < 0.001, respectively. Values given are means ± 1 standard error.

**Figure 6:**
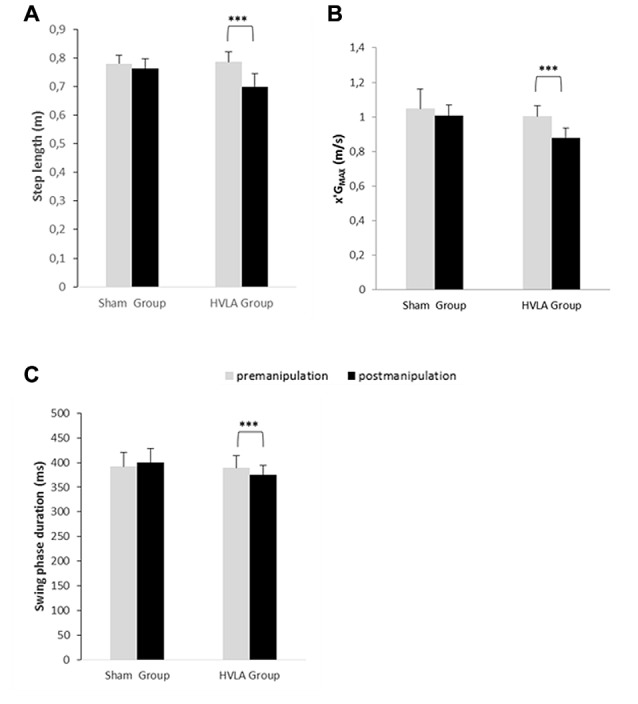
Comparison of swing phase-related parameters of gait initiation between the pre- and post-manipulation conditions in the sham and HVLA groups. **(A)** Step length, **(B)** x′G_MAX_: peak center of gravity velocity and **(C)** swing phase duration. *** Statistical difference with *p* < 0.01, *p* < 0.001, respectively. Values given are means ± 1 standard error.

The *post hoc* analysis further indicated that, for the HVLA group, each of the gait initiation variables investigated in this study was significantly lower in the post-manipulation condition than in the pre-manipulation condition (see Figures 5, 6 for details on the *post hoc* analysis). In contrast, regarding the sham group, none of these variables significantly differed between the pre- and post-manipulation condition. Finally, it is noteworthy that none of the gait initiation variables significantly differed between the sham and the HVLA groups in the pre-manipulation condition. The two groups were therefore homogeneous with respect to these variables before the manipulation.

## Discussion

This study investigated the effect of SMT-HVLA manipulation applied to T9 on spine ROM and on the biomechanical organisation of gait initiation. Participants purposely performed series of gait initiation trials before and after sham or SMT-HVLA manipulations. Spine ROM and classical biomechanical gait initiation parameters were compared in the pre- and post-manipulation conditions.

The results showed that the spine ROM, and especially the maximal thoracic flexion, was larger post-manipulation than pre-manipulation in the HVLA group, which was not the case in the sham group. The mean increase was 20%. The HVLA manipulation applied to T9 had, therefore, a beneficial effect on spine mobility, even in a group composed of young adults with no known spine pathologies. A similar result was found in the sole study to date that tested the effect of thoracic HVLA manipulation on spine ROM (Schiller, 2001). However, this study only examined right and left thoracic lateral flexion using a goniometer (BROM 2), and the population tested included patients with thoracic back pain. To our knowledge, the other studies focusing on thoracic HVLA measured the cervical ROM, and reported small or no beneficial effects (González-Iglesias et al., 2009; Suvarnnato et al., 2013). These negligible variations have led us to exclude the cervical section from spine ROM measurement.

SMT-HVLA has been shown to have a beneficial effect on spine-related pain, both clinically and in experimentally-induced pain (for reviews see Millan et al., 2012a,b). However, it remains unclear from the literature if it has an immediate noticeable biomechanical effect on spinal motion that can be measured in terms of an increased ROM (Millan et al., 2012a). One of the main goals of healthcare practitioners applying SMT-HVLA manipulation is to increase spine ROM, e.g., in athletes before competition or in patients with spine stiffness. A recent review on this aspect emphasized that some studies found spinal manipulation to have limited effect on the ROM, while others found it had none (Millan et al., 2012a). These mitigated effects could probably stem from many factors such as the different tools used in research and in clinical practice to measure ROM (single/double/triple inclinometers, goniometers, a rangiometer, tape measures, visual estimation, spine motion analyzer, etc.), the direction, duration and force applied to the participant’s spine, the expertise of the practitioner etc. The present study shows that analogical inclinometers may be used to detect an increased spine ROM following thoracic SMT-HVLA.

Postural mobility, and especially spine mobility, is known to be a key factor of the PKC (Bouisset and Zattara, 1987; Bouisset and Do, 2008). According to the PKC concept, any factor that may enhance (or conversely, alter) this capacity would favor (or hinder) the motor performance and postural stability. This concept has been substantiated by many recent experimental studies which manipulated spine mobility using various means, e.g., by the application of splints at different levels of the spine (Alamini-Rodrigues and Hamaoui, 2016), by experimentally-induced trunk muscular tension (Hamaoui et al., 2004, 2011; Hamaoui and Le Bozec, 2014), or by changing the contact surface between the thighs and seat in the sitting posture (Lino et al., 1992; Teyssèdre et al., 2000; Le Bozec and Bouisset, 2004; Diakhaté et al., 2013). It has been shown that constraining spine mobility may disturb postural equilibrium when maintaining erect posture as revealed by measuring the COP. In dynamical tasks, such as sit-to-stand (Diakhaté et al., 2013; Alamini-Rodrigues and Hamaoui, 2016), maximal isometric ramp push (Le Bozec and Bouisset, 2004), arm pointing (Lino et al., 1992; Teyssèdre et al., 2000) or trunk flexion (Diakhaté et al., 2013) from the sitting posture, facilitating spine mobility has been shown to favor APA development and thus motor performance. Based on the results of these studies—and given that spine mobility is known to be highly solicited during locomotion and gait initiation (e.g., Thorstensson et al., 1984; Ceccato et al., 2009; Cusin et al., 2017), APA development and motor performance could have been expected to be facilitated following SMT-HVLA. It is also noteworthy that T9 is described as the “walking vertebra”, a concept arising from the classical article of Wernham (1985). This concept is based on the fact that the T9 vertebra is the inflexion point of the curvature change of thoracic cyphosis in lumbar lordosis. This vertebra ensures the junction of the thoracic and lumbar segments, mainly in their counter-rotation movement, especially while walking. As such, the T9 HVLA manipulation is commonly used by healthcare practitioners in patients with locomotor deficiencies. However, its impact on the locomotor function has to date never been evaluated in systematic studies. In contrast to our expectations, APA amplitude and duration decreased following manipulation in the HVLA group, by 24% and 6%, respectively. This was not the case in the sham group, which shows that this result could not be ascribed to a placebo effect. Not only were the APA parameters reduced, but their efficiency (computed as the ratio [COG velocity at foot-off/APA duration]) was reduced (by 23% as compared to the pre-manipulation condition). In other words, the capacity of the postural system to generate forward propulsive forces during the limited duration of APA was less efficient post-manipulation. As a consequence of the lower initial (foot-off) COG velocity, the peak COG velocity (speed performance) and step length both reached lower values post-manipulation in the HVLA group (compared to the pre-manipulation condition, the decrease was 14%, 12% and 11%, respectively). This finding was expected since it is well-known that the two latter step parameters are positively correlated with the amplitude of APA, i.e., the higher the peak anticipatory backward COP shift is, the higher the speed performance and step lengths are (Brenière et al., 1987). Because there was no change in APA parameters post-manipulation in the sham group, step length and speed performance remained the same as in the pre-manipulation condition. Because in the present study, spine mobility was increased following the HVLA manipulation, which is known to be a factor of improved motor performance, the question arises as to why APA development and speed performance were impaired instead of being improved.

Besides its mechanical effect on spine mobility (for reviews see Pickar, 2002; Millan et al., 2012a), SMT-HVLA is known to induce transient changes in the sensorimotor pathways and structures involved in the coordination between posture and movement. As stressed in the “Introduction” Section, studies using the H-reflex technique to investigate the effect of SMT-HVLA on motoneuronal excitability reported controversial findings, i.e., both an increased (Niazi et al., 2015) and a decreased excitability (Murphy et al., 1995; Dishman and Burke, 2003; Suter et al., 2005) have been found. It seems however that there exists a consensus concerning the effects of spinal manipulation on the sensorimotor processing and integration at the cortical level, as evidenced with the somatosensory evoked potential technique (SEP). Specifically, recent studies reported an alteration of the amplitude of the cortical SEP peaks N20 and N30 following SMT-HVLA (Haavik and Murphy, 2012; Lelic et al., 2016). The N20 peak is known to represent the arrival of the afferent volley at the primary somatosensory cortex (Desmedt and Cheron, 1980; Nuwer et al., 1994; Mauguière, 1999), while later peaks such as the N30 SEP peak are thought to reflect early sensory-motor integration (Rossi et al., 2003; regarding the possible generators of this peak, see Haavik and Murphy, 2012; Lelic et al., 2016). In addition, studies in the anesthetized cat have shown that spinal manipulation induced changes in the discharge of mechanoreceptors from the paraspinal region, especially group Ia spindle afferents (Pickar, 2002; Reed et al., 2015). The extent to which the cortical and afferent responses to spinal manipulation reflect beneficial outcomes (e.g., pain relief), remains largely unclear in the literature; however, what is clear, is that these responses have the potential to induce changes in the coordination between posture and movement, which is known to strongly rely on proprioceptive inputs from the postural limbs, and even more importantly, on how the CNS processes, interprets and transforms this afferent information into motor commands (Paulus and Brumagne, 2008; Haavik and Murphy, 2012). Lelic et al. (2016) recently speculated that since spinal manipulation is known to reduce pain and improve function in clinical trials (Botelho and Andrade, 2012; Mieritz et al., 2014; Schneider et al., 2015), the observed reduction of the N30 amplitude following SMT-HVLA may reflect a beneficial change. However, it should be noted (as the authors did) that reduced N30 SEP peak amplitudes have also been found in the literature in pathological populations such as Parkinson’s disease (Cheron et al., 1994; Kang and Ma, 2016), known to have deficits in APA production during both voluntary lower (e.g., Delval et al., 2014) and upper limb tasks (e.g., Bazalgette et al., 1987). Kang and Ma (2016) even reported that frontal N30 status indicated the motor severity of Parkinson’s disease. During gait initiation, disturbances in Parkinson’s disease include reduced APA and abnormal APA timing (Delval et al., 2014). During arm elevation, postural movements are known to be less anticipatory in Parkinson’s patients than in controls (Bazalgette et al., 1987). In the present study, APA were also less anticipatory, had a smaller amplitude and were less efficient in the HVLA group following manipulation than prior to manipulation. Globally taken, the results from the literature may thus suggest that a reduction of the N30 amplitude after HVLA manipulation may reflect a transitory alteration in the cortical integration of sensory-motor information, and may thus reflect a negative change. If so, such alteration has the potential to affect motor coordination during locomotor tasks such as gait initiation. In other words, we propose that a neural effect, possibly mediated by a transient alteration in the early sensory-motor integration following SMT-HVLA could be one of the mechanisms responsible for the present results. This neural effect may have masked the potential mechanical benefits associated with increased spine mobility.

## Study Limitations

There are some limitations to the present study that should be pointed out. First, this study only focused on a biomechanical investigation. It is clear that studies linking the changes in motor behavior observed in the present study, to the changes of activity in the neural structures and pathways reported in the literature should be carried out to further substantiate the data interpretation. This is why we used the term “pilot” in the title of this article. Second, it should be emphasized that only short-term effects were investigated. It is not excluded that thoracic HVLA manipulation may have a long-term beneficial effect on APA and speed performance. Third, the biomechanical responses described in this study were obtained from young healthy participants and may not be generalizable to other populations, including patients with spinal pain. Finally, it is known that a manipulation is rarely specific to only the adjustment site (Ross et al., 2004). This non-specificity is amplified by the technique used in this article as it is an indirect technique. There is no direct contact of the practitioner with the chosen vertebra since the compressive force is indirectly transmitted by the hand of the patient between his own vertebrae and the thorax of the practitioner. We point out that the role of T9 vertebra and the interest of its manipulation is based solely on empirical knowledge although these notions are still taught in physiotherapy and osteopathy schools. Currently, some studies suggest that the center of rotation of the thoracic zone in the horizontal plane corresponds to a very wide area (T7–L3; Konz et al., 2006).

## Conclusion

The present results showed that thoracic HVLA manipulation in young healthy participants has an immediate beneficial effect on spine mobility but a detrimental effect on APA development and speed performance during gait initiation. It thus seems that HVLA manipulation should be considered with caution by participants who seek an immediate increase of speed performance during locomotor tasks.

## Author Contributions

SD and EY designed the study; collected, analyzed and interpreted the data; drafted and revised the manuscript; gave final approval. AH and AD interpreted the data; drafted and revised the manuscript.

## Conflict of Interest Statement

The authors declare that the research was conducted in the absence of any commercial or financial relationships that could be construed as a potential conflict of interest. The handling Editor currently co-hosts a Research Topic with one of the authors EY, and confirms the absence of any other collaborations. He states that the process met the standards of a fair and objective review.
